# Transcranial Doppler Pulsatility Index and MRI Findings in Meningoencephalitis: A Pilot Observational Retrospective Cohort Study in Critically Ill Patients

**DOI:** 10.3390/clinpract16020041

**Published:** 2026-02-14

**Authors:** Maria Grazia Bocci, Giulia Capecchi, Antonio Lesci, Dorotea Rubino, Ilaria Caravella, Giorgia Taloni, Valerio Sabatini, Candido Porcelli, Giulia Valeria Stazi, Gabriele Garotto, Elena Mattiucci, Emanuele Nicastri, Tommaso Ascoli Bartoli, Gaetano Maffongelli, Emiliano Cingolani, Fabrizio Albarello, Giulia Anello, Paolo Campioni, Stefania Ianniello, Daniele Guerino Biasucci

**Affiliations:** 1 Clinical and Research Department, National Institute for Infectious Diseases “Lazzaro Spallanzani”—IRCCS, 00149 Rome, Italy; mariagrazia.bocci@inmi.it (M.G.B.); dorotea.rubino@inmi.it (D.R.); ilaria.caravella@inmi.it (I.C.); giorgia.taloni@inmi.it (G.T.); valerio.sabatini@inmi.it (V.S.); candido.porcelli@inmi.it (C.P.); giuliavaleria.stazi@inmi.it (G.V.S.); gabriele.garotto@inmi.it (G.G.); elena.mattiucci@inmi.it (E.M.); 2 Istituto di Anestesiologia e Rianimazione, Università Cattolica del Sacro Cuore, 00161 Rome, Italy; antonio.lesci01@icatt.it; 3 Clinical and Research Infectious Diseases Department, National Institute for Infectious Diseases, Lazzaro Spallanzani—IRCCS, 00149 Rome, Italy; emanuele.nicastri@inmi.it (E.N.); tommaso.ascoli@inmi.it (T.A.B.); gaetano.maffongelli@inmi.it (G.M.); 4 Anesthesia and Intensive Care, San Camillo-Forlanini Hospital, 00152 Rome, Italy; ecingolani@scamilloforlanini.rm.it; 5 Radiology Unit, National Institute for Infectious Diseases “Lazzaro Spallanzani”—IRCCS, 00149 Rome, Italy; fabrizio.albarello@inmi.it (F.A.); giulia.anello@inmi.it (G.A.); paolo.campioni@inmi.it (P.C.); stefania.ianniello@inmi.it (S.I.); 6 Department of Clinical Science and Translational Medicine, ‘Tor Vergata’ University of Rome, 00133 Rome, Italy; biasucci@med.uniroma2.it

**Keywords:** transcranial color-coded Doppler, neuromonitoring, neuroinflammation meningoencephalitis, critical care, pulsatility index, MRI

## Abstract

**Background:** Meningoencephalitis is a complex inflammatory condition of the CNS that can result in significant morbidity and mortality in critically ill adults. Accurate and timely neuromonitoring is essential for guiding management and improving outcomes. This study aimed to descriptively evaluate the prognostic value of early TCCD monitoring, particularly the pulsatility index, and its integration with conventional and perfusion MRI in patients with meningoencephalitis. **Methods:** We present an observational, retrospective, cohort study involving ten adult patients (median age 56 years, IQR 45.5–68.5; mean 55.9, range 35–76) with neurological syndromes caused by suspected or confirmed infectious meningoencephalitis. Etiologies included bacterial meningitis/meningoencephalitis (50%), viral meningoencephalitis (10%), neurotoxoplasmosis (10%), progressive multifocal leukoencephalopathy (10%), and undetermined origin (20%). Patients underwent TCCD and MRI within 24 h. In five cases, standard MRI sequences were acquired, while in the remaining five, perfusion imaging was performed using Arterial Spin Labelling (ASL). A favorable outcome was defined as survival with neurological recovery (Glasgow Outcome Scale > 5) at ICU discharge. **Results:** TCCD-derived PI provided valuable information on cerebral hemodynamics. PI values ≤ 1.25 were associated with favorable clinical outcomes and symmetrical MRI findings. Conversely, PI > 1.25 correlated with poor prognosis and often preceded MRI-detectable structural damage. When combined with ASL, PI mirrored the detected perfusion asymmetries and was associated with poor prognosis in fatal cases. **Conclusions:** Bedside TCCD can offer real-time assessment of cerebrovascular dynamics and, when integrated with conventional and ASL MRI, could enhance the understanding of pathophysiological processes in meningoencephalitis, supporting timely and informed decisions in neurocritical care.

## 1. Introduction

Meningitis and encephalitis are inflammatory syndromes of the central nervous system that frequently require intensive care due to the risk of rapid neurological deterioration. While meningitis primarily affects the meningeal layers, encephalitis involves parenchymal inflammation, often manifesting with encephalopathy, seizures, and elevated intracranial pressure (ICP). When both components coexist, defined as meningoencephalitis, diagnostic and prognostic challenges become particularly complex [1,2].

In such a context, multimodal neuromonitoring plays a central role [3]. However, conventional methods, such as repeated CT or invasive ICP catheters, are limited by either logistical constraints or risks in unstable patients. In recent years, Transcranial Color-Coded Doppler (TCCD) has emerged as a valuable, non-invasive tool capable of providing continuous, real-time bedside assessment of cerebral hemodynamics, including estimations of cerebrovascular resistance, perfusion symmetry, potential surrogates of ICP and autoregulation [4,5].

When combined with magnetic resonance imaging (MRI), TCCD provides a unique opportunity to merge functional and structural information. Sequences such as FLAIR and diffusion-weighted imaging (DWI) allow early visualization of inflammation, edema, or infarction [6,7,8]. Perfusion sequences, particularly Arterial Spin Labelling (ASL), offer quantitative evaluation of cerebral blood flow (CBF) without contrast, complementing TCCD hemodynamic findings with spatial resolution [9,10].

Despite its widespread application in acute traumatic and non-traumatic brain injury, the use of TCCD in meningoencephalitis remains underreported. Moreover, the integration of TCCD parameters with MRI-based structural and perfusion data to improve prognostication is not yet standardized. In this study, we describe ten critically ill adult patients with meningoencephalitis who underwent both TCCD and MRI (standard or perfusion), and we analyze how combined interpretation can inform prognosis and guide management in the ICU setting. Our study aimed to provide a first descriptive account of pulsatility index (PI) obtained by TCCD examination in patients with meningoencephalitis, and to explore its relationship with MRI features. While preliminary and hypothesis- generating, our data suggest that TCCD might have potential as a non-invasive bedside tool to complement neurological examination and imaging in the acute phase. This is a step toward developing more targeted monitoring strategies that could ultimately lead to improved patient outcomes.

## 2. Methods

### 2.1. Study Design and Setting

This was a small observational, retrospective, cohort study conducted in the Intensive Care Unit (ICU) of the National Institute for Infectious Diseases “Lazzaro Spallanzani” (IRCCS), Rome, Italy. The study retrospectively included ten adult patients (age > 18 years) admitted between January and December 2024 with neurological syndrome caused by suspected or confirmed infectious meningoencephalitis. Patients were included if transcranial Doppler (TCCD) monitoring had been performed within 48 h of hospital admission.. Meningoencephalitis was defined as depressed or altered mental status, neurological deterioration (GCS < 9), lethargy, or personality changes together with at least two of the following criteria: fever (≥38 °C) or hypothermia (≤35 °C); cerebrospinal fluid (CSF) pleocytosis (≥5 cells/mm^3^) or pathogen isolation; radiological findings consistent with meningoencephalitis; focal neurological deficits; meningismus; electroencephalography (EEG) abnormalities compatible with encephalitis; or seizures. Cases were classified as suspected when based on clinical and/or CSF alterations without microbiological confirmation, and as confirmed when a pathogen was isolated from CSF. The study did not include the entire cohort of patients admitted to the ICU of the National Institute for Infectious Diseases “Lazzaro Spallanzani” with meningoencephalitis, but only those who, at the time of submission, had undergone both TCCD and MRI, including MRI-CASL sequences. This subgroup therefore represents the cases for which both examinations were available and suitable for analysis.

### 2.2. Ethical Approval

Ethical review and approval were waived for this study by the Ethics Committee (Comitato Etico Territoriale Lazio Area 4; protocol code: 2C-2025; approval date: 29 January 2025). Due to its retrospective nature, informed consent was waived. The study adhered to the principles of the Declaration of Helsinki and to the Good Clinical Practice Guidelines.

### 2.3. Patient Selection and Grouping

All included patients underwent bedside neuromonitoring with Transcranial Color-Coded Doppler (TCCD) within 48 h of clinical neurological deterioration requiring ICU admission. This was a non-consecutive sample restricted to patients who underwent both TCCD and MRI (and ASL in a subset) within the defined time window; therefore, selection bias is expected. Patients were stratified into two groups based on the type of brain MRI received:

- Group 1: TCCD + standard MRI with FLAIR and Diffusion-Weighted Imaging (DWI)
- Group 2: TCCD + perfusion MRI using Arterial Spin Labelling (ASL), in particular CASL (continuous arterial spin labelling)

In all cases, clinical data, TCCD parameters, MRI results, outcomes at day 28 and a follow-up at 3 months were collected and analyzed.

MRI and TCCD assessments were performed within 24 h to ensure temporal consistency between imaging and ultrasonographic measurements.

### 2.4. Transcranial Color-Coded Doppler (TCCD)

TCCD was performed using a 2–2.5 MHz sectorial transducer applied through the transtemporal acoustic window [4]. Standard insonation planes (mesencephalic, diencephalic, and ventricular) were acquired bilaterally [5]. The middle cerebral artery (MCA) was identified at a depth of 30–60 mm using color-coded duplex imaging [4]. Mean flow velocities (MFV), systolic and diastolic velocities, and pulsatility index (PI) were measured and registered for both MCAs. PI, calculated as (PSV − EDV)/MFV, allows for assessment of cerebrovascular hemodynamics and for eventual detection of high ICP [11,12,13,14,15].

PI values and thresholds were interpreted and presented descriptively. PI values ≤ 1.25 were considered normal and indicative of adequate cerebral perfusion, whereas PI values > 1.25 were regarded as suggestive of perfusion deficits. The 1.25 value was used as a pragmatic reference point, but cut-offs were not prospectively defined for decision-making and were not statistically derived in this cohort. Higher PI values (e.g., >1.65) are reported as observed associations within this pilot sample and should not be interpreted as validated thresholds. 

The following parameters were recorded for each patient during the TCCD diagnostic assessment phase: age, sex, hematocrit (Hct), arterial PaCO_2_, sedation and mean arterial pressure (MAP). Overall, all insonation windows were adequate, and no angle correction was required.

### 2.5. Magnetic Resonance Imaging (MRI)

All MRIs were performed using a 1.5 Tesla scanner, the SIGNA™ Voyager 1.5T, manufactured by GE HealthCare (Chicago, IL, USA).

Sequences included:

- FLAIR (Fluid-Attenuated Inversion Recovery): to detect lesions with high contrast against suppressed CSF
- DWI (Diffusion-Weighted Imaging): to identify cytotoxic edema and acute infarcts
- ADC (Apparent Diffusion Coefficient): to quantify water molecule motion, aiding differentiation between acute ischemia and other pathologies.

Perfusion MRI (ASL) Protocol. ASL was used in Group 2 to assess non-invasively regional cerebral blood flow (CBF) in mL/100 g tissue/min. ASL is an MRI technique that uses magnetized water molecules in blood cells as an endogenous tracer. This methodology provides images proportional to brain perfusion and CBF, characterized by good spatial resolution. In this study, Continuous ASL (CASL) was employed. A continuous labeling pulse (2–4 s) was applied at the base of the skull with a slice-selective gradient to invert arterial spins flowing toward the brain. After a post-labeling delay, optimized for adult cerebral circulation, these inverted spins reached the target tissue, producing a perfusion-weighted signal. Labeling efficiency, which depends on flow velocity, vessel angle relative to the labeling plane, pulse amplitude, and gradient strength, was incorporated into perfusion quantification. To minimize magnetization transfer (MT) effects, control images were acquired using pulses matched to the label condition but applied in the opposite direction, ensuring MT-related signal changes were present in both acquisitions and cancelled upon subtraction. The resulting perfusion-weighted images underwent post-processing to generate parametric color maps of regional cerebral perfusion. Quantitative CBF values were extracted by placing regions of interest (ROIs) in predefined brain areas.

### 2.6. Clinical Outcome

Functional outcome at 28 days was assessed using the Glasgow Outcome Scale (GOS), with the score GOS = 5 considered favorable recovery. Mortality at day 28 was also recorded. Patient follow-up was performed at 3 months, and their status was assessed using the Glasgow Outcome Scale-Extended (GOS-E) with a GOS-E score = 8 considered as upper good recovery.

### 2.7. Statistical Analysis

Due to the retrospective and descriptive nature of this study, no formal statistical analysis or sample size calculation was performed. The findings are based on clinical observations and qualitative correlations between neuromonitoring parameters and patient outcomes. Only descriptive statistics were performed for demographic and clinical variables. Categorical data were expressed as absolute and relative frequencies, while continuous variables were summarized as mean, median, interquartile range, and range. As an exploratory analysis, in the subgroup of patients who underwent CASL, the association between mean pulsatility index (PI) and percent cerebral blood flow (CBF) asymmetry was assessed using Spearman’s rank correlation coefficient, chosen due to the very small sample size and the exploratory nature of the analysis.

## 3. Results

A total of ten patients were included in the analysis. The median age was 56 years (IQR 45.5–68.5), with a mean age of 55.9 years (range 35–76). Six patients (60%) were female and four (40%) were male (Table 1).

Regarding etiology, five cases (50%) were due to bacterial meningitis/meningoencephalitis, one (10%) to viral meningoencephalitis, one (10%) to neurotoxoplasmosis, one (10%) to progressive multifocal leukoencephalopathy (PML), and two (20%) to meningoencephalitis of undetermined origin.

**Figure 1:**
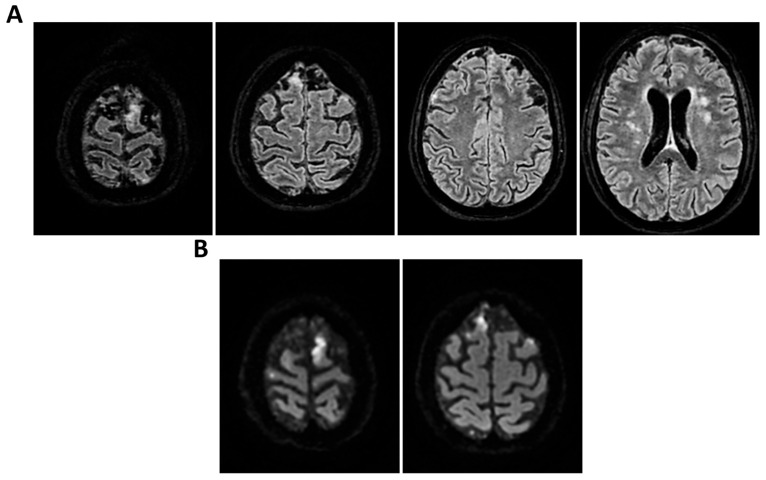
**MRI of patient 1 group 1.** A 74-year-old woman with a history of atrial fibrillation, ischemic heart disease, anxiety-depressive syndrome, and hypertension was found at home in a stuporous state. She had experienced asthenia and fever in the preceding days and had initiated ciprofloxacin treatment. On arrival, laboratory exams showed leukocytosis (11,930/mm^3^, 90.4% neutrophils), elevated INR (1.92), and markedly increased inflammatory markers. Arterial blood gas revealed hypoxemia (pO_2_ 55 mmHg), requiring mechanical ventilation with 35% FiO_2_. Brain CT was unremarkable, showing no acute lesions or signs of raised intracranial pressure. Chest CT revealed bilateral posterior-basal consolidations with air bronchograms (more severe on the left), a smaller consolidation in the left upper lobe, and bilateral pleural effusions, consistent with bronchopneumonia. A lumbar puncture was initially contraindicated due to coagulopathy. Due to worsening neurological status (GCS 6), the patient was transferred to the ICU, intubated, and underwent lumbar puncture. On day 2, TCCD showed PI values of 0.62 (right) and 0.71 (left), with no signs suggestive of intracranial hypertension. (**A**) 3D axial FLAIR sequence shows multiple bilateral hyperintense areas, without mass effect. (**B**) Some of these present diffusion restriction in axial DWI sequence with the largest located in the left middle and superior frontal gyrus. There is also a subtle pachymeningeal enhancement in the bilateral fronto-temporal region. Supratentorial and infratentorial ventricular system is normal in size and configuration, with no midline shift or hydrocephalus.

**Figure 2:**
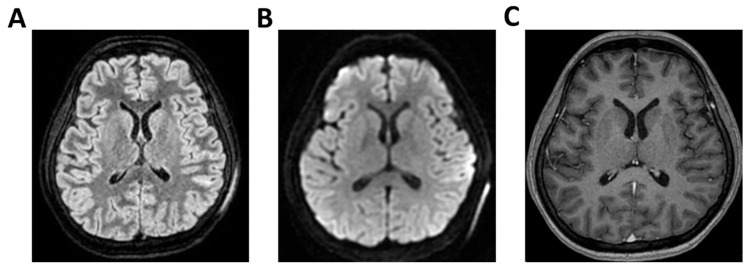
**MRI of patient 2 group 1.** A 37-year-old woman presented to the emergency room (ER) with acute confusion and fever that began earlier the same day. Neurological evaluation and brain CT were unremarkable, as was chest CT. A lumbar puncture was performed. FilmArray was negative for bacteria but showed elevated cell count. Immunoglobulins for *Clostridium tetani* were administered empirically. The patient developed generalized myoclonic movements. EEG revealed epileptiform activity in the right frontotemporal region and diffuse cerebral slowing. Due to worsening neurological status, she was intubated and transferred to the ICU. On day 2 of ICU admission, transcranial Doppler TCCD showed pulsatility indices of 0.94 (left) and 0.89 (right), with no evidence of intracranial hypertension. (**A**) 3D axial FLAIR, (**B**) axial DWI and (**C**) 3D axial T1 FSPGR after administration of contrast medium show normal morphology and signal intensity of brain tissue. There is no evidence of acute or chronic infarction, demyelination or mass lesion. Supratentorial and infratentorial ventricular system is normal in size and configuration, with no midline shift or hydrocephalus. No signs of intracranial hypertension. Sulci and subarachnoid spaces are within normal limits for age.

**Figure 3:**
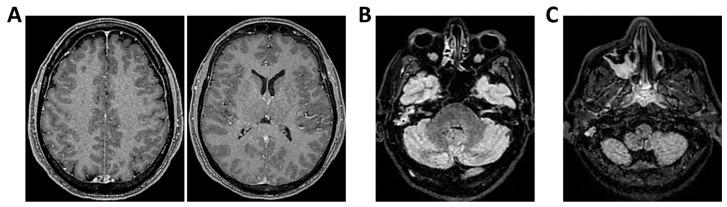
**MRI of patient 3 group 1.** A 49-year-old man with pneumococcal meningitis was transferred to INMI L. Spallanzani after 10 days of right-sided otalgia, fever, and headache. On arrival, he was alert and oriented but exhibited psychomotor slowing. Physical exam revealed positive Kernig’s, Brudzinski’s, and Lasegue’s signs. Brain CT was negative for acute lesions but showed bilateral maxillary sinusitis and right-sided otomastoiditis. Lumbar puncture revealed cloudy CSF with 4069 cells/mm^3^, low glucose (36 mg/dL), and elevated protein (180 mg/dL). FilmArray was positive for *Streptococcus pneumoniae*; urinary antigens for *Legionella pneumophila* and *S. pneumoniae* were negative. The patient was started on Vancomycin, Ceftriaxone, and Dexamethasone. The next day, he became mute and poorly cooperative, prompting transfer to the ICU. TCCD showed normal PI (1.18 right, 1.02 left) with no signs of intracranial hypertension. (**A**) 3D axial T1 FSPGR (Fast Spoiled Gradient Recalled) shows mild and diffuse increase in physiological pachymeningeal enhancement, with no specific significance. Morphology and signal intensity of brain tissue is normal. (**B**) 3D axial FLAIR confirms right-sided otomastoiditis and (**C**) bilateral maxillary sinusitis.

**Figure 4:**
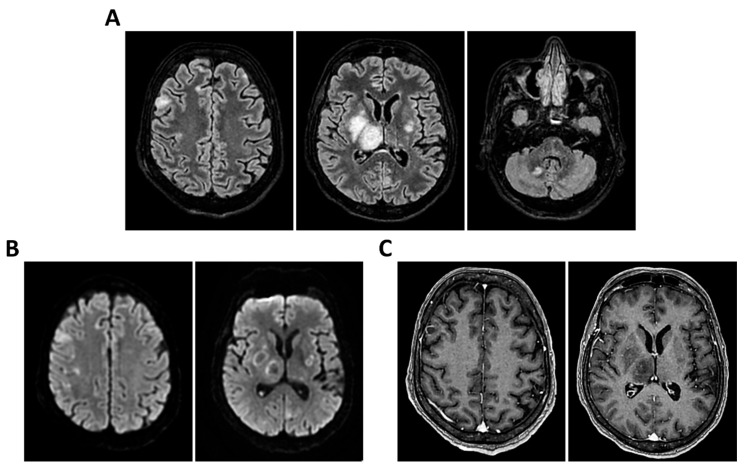
**MRI of patient 4 group 1.** A 46-year-old HIV-positive woman with a history of substance abuse, alcohol use, heavy smoking, and on methadone therapy was admitted to the ER after a fall with head trauma. Cranial CT revealed a hypodense, heterogeneous lesion in the right nucleus-capsular region. Chest CT showed pseudonodular lesions, parenchymal consolidations (lingular and posterior-basal left lung), bilateral fibrous-scar tissue thickening, and polylobulated thickening in the right upper lobe. Due to respiratory deterioration, she was intubated and admitted to the ICU. Infectious disease consultation revealed severe immunosuppression (CD4 count 37/mm^3^), high HIV and HCV viral loads, and positive toxoplasmosis serology. She was not on antiretroviral therapy. Transferred to a specialized ICU, she was diagnosed with neurotoxoplasmosis. On day 2, TCCD showed altered CBF with PI of 1.96 on the right and 1.25 on the left, indicating possible intracranial hypertension. (**A**) 3D axial FLAIR sequence shows multiple bilateral supratentorial and infratentorial hyperintense areas. (**B**) Some of these areas present diffusion restriction in axial DWI sequence with the largest located in the right basal ganglia with moderate compression on the third ventricle. (**C**) One of these areas in the right middle frontal gyrus shows “ring enhancement” in the 3D axial T1 FSPGR after administration of contrast medium.

**Figure 5:**
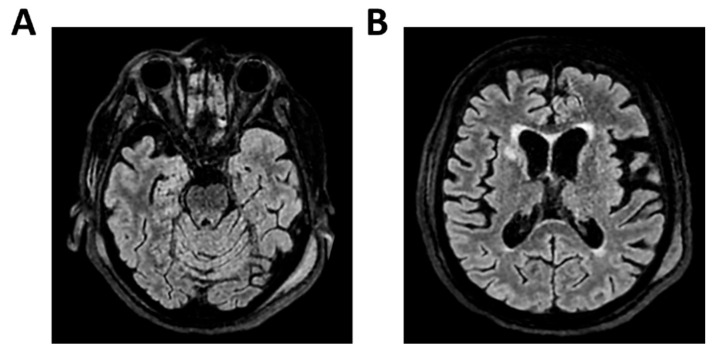
**MRI of patient 5 group 1.** A 76-year-old diabetic woman was admitted to the ER for confusion, slurred speech, hyperglycemia, dyspnea (SpO_2_ 87% on room air), and recurrent syncopal episodes, the latest resulting in a fall with frontal head trauma. Brain CT and neck vessel angio-CT were negative for acute lesions. Chest CT revealed a D11 vertebral collapse (post-vertebroplasty), right rib fractures (4th–7th), and right basal pleural effusion with ventilation impairment. During hospitalization, she developed fever and a right facial rash suggestive of herpes zoster. Empiric therapy with Tazocin and Zovirax was started, later broadened to include Rocephin and Ampital. On day 4, the patient experienced a decline in consciousness, necessitating intubation and mechanical ventilation. CSF analysis via lumbar puncture was positive for Varicella Zoster virus (FilmArray). She was transferred to a specialized ICU. On the second day in the ICU, TCCD showed severe CBF alteration, with a PI of 3.6 and markedly impaired flow in the left MCA, indicating likely intracranial hypertension and poor perfusion. (**A**) 3D axial FLAIR sequence shows subtle soft hyperintensity in the right hippocampal region likely due to inflammatory phenomena. (**B**) No diffusion restriction in DWI or contrast enhancement, some small areas of hyperintensity in the bilateral corona radiata and centrum semiovale, suggestive of chronic vascular injury and soft tissue swelling in the left parieto-occipital region.

**Figure 6:**
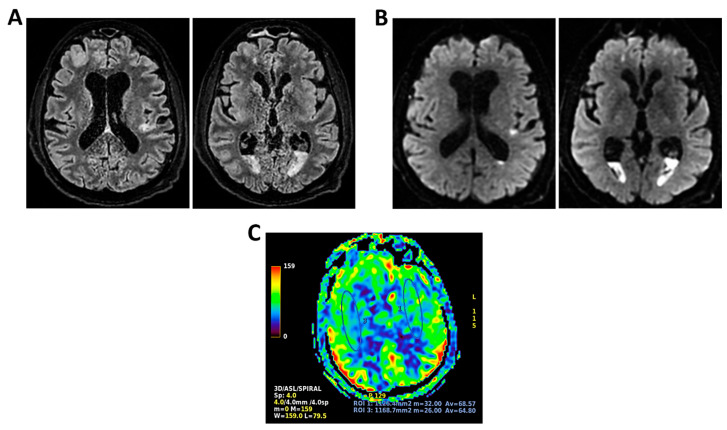
**MRI of patient 1 group 2.** A 63-year-old man with a history of hypertension, dyslipidemia, and prior pulmonary lobectomy was admitted to the ER in a drowsy but verbally responsive state. His wife reported a recent episode of vomiting followed by loss of consciousness and tonic–clonic seizures. He was uncooperative and tremulous but afebrile, although a febrile episode had occurred in the preceding days. A lumbar puncture led to a diagnosis of *Neisseria meningitidis* type B meningitis. Ceftriaxone 2 g IV every 12 h was initiated. Brain CT, chest CT, and angio-CT of the epiaortic vessels showed no acute abnormalities. Due to clinical deterioration and a further seizure with hypotension, the patient was sedated, intubated, and experienced an episode of ventricular tachycardia during CT imaging, requiring IV Amiodarone. The patient was transferred to the ICU. On day 2, TCCD showed no significant flow abnormalities, with only mild asymmetry in PI (1.11 right, 1.4 left), without evidence of intracranial hypertension. (**A**) MRI exam with perfusion study reveal areas of altered signal intensity, hyperintense on 3D axial FLAIR sequence, (**B**) with diffusion restriction in axial DWI sequence, indicative of subacute inflammatory processes. These areas were scattered, the most significant is located along the subpial cortical surfaces of the left precentral and supramarginal gyri. Additionally, MRI exam demonstrates leveled material of likely inflammatory nature in both occipital horns of the lateral ventricles (**A**,**B**). (**C**) MRI perfusion with ASL sequence shows normal and symmetrical CBF values. Blue circles indicate the region of interest (ROI) used for quantitative CBF analysis.

**Figure 7:**
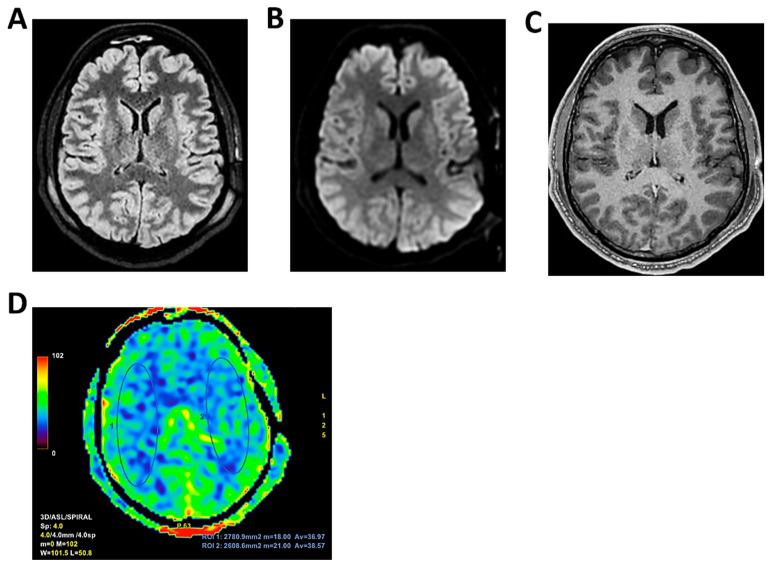
**MRI of patient 2 group 2.** A 45-year-old male without prior clinical conditions experienced chills and general discomfort and dizziness. He arrived at the ER unresponsive with a GCS score of 10. A brain CT scan showed no signs of hemorrhage or thrombosis, and a lumbar puncture was carried out for microbiological and biochemical analysis. Empirical therapy with Rocephin, Zovirax, and Ampicillin was initiated, and both neurology and intensive care consultations were obtained. Given the likely progressive clinical condition, the patient was sedated, intubated, and transferred to the ICU for further treatment. On the second day, the patient underwent a TCCD, which documented a PI of 1.32 on the right and 1.28 on the left (**A**) 3D axial FLAIR, (**B**) axial DWI and (**C**) 3D axial T1 FSPGR after administration of contrast medium demonstrate normal morphology and signal intensity of brain tissue. No evidence of acute or chronic infarction, demyelination or mass lesion. Supratentorial and infratentorial ventricular system is normal in size and configuration, with no midline shift or hydrocephalus. No signs of intracranial hypertension. Sulci and subarachnoid spaces are within normal limits for age. (**D**) MRI perfusion with ASL sequence shows normal and symmetrical CBF values. Blue circles indicate the ROI used for quantitative CBF analysis.

**Figure 8:**
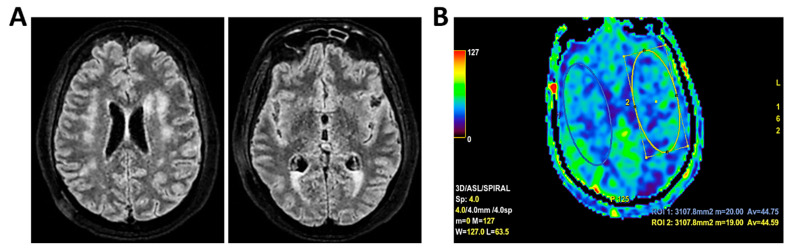
**MRI of patient 3 group 2.** A 67-year-old woman with hypertension, depressive disorder, and hypothyroidism was brought to the ER in a confused state, following three days of abdominal pain, fever, neck pain, and headache. On arrival, she was drowsy but responsive to verbal stimuli and able to follow simple commands. Total-body CT showed no cranial or abdominal pathology except for a 6 cm rectal fecaloma; lungs were clear. A lumbar puncture revealed cloudy CSF with 1100 cells/mm^3^. FilmArray was positive for *S. pneumoniae*. She was treated with Rocephin and Tazocin and transferred to our ICU. On day 2, TCCD showed no flow abnormalities, with PI values of 1.2 (right) and 1.1 (left). (**A**) 3D axial FLAIR sequence shows multiple hyperintense areas, without significant diffusion restriction in axial DWI sequence, localized in bilateral corona radiata and centrum semiovale and leveled material of likely inflammatory nature in both occipital horns of the lateral ventricles. (**B**) The perfusion study revealed normal and symmetrical CBF values. Blue and yellow circles indicate the ROI used for quantitative CBF analysis.

**Figure 9:**
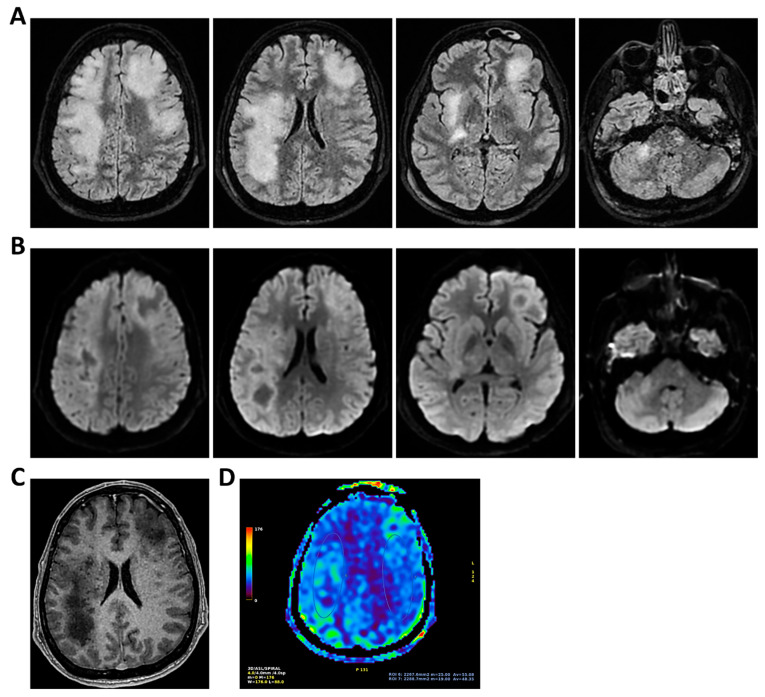
**MRI of patient 4 group 2.** A 35-year-old HIV-positive man, diagnosed in 2018 with pneumocystosis (PCP) and non-compliant with Highly Active AntiRetroviral Therapy (Biktarvy), presented with bilateral otalgia, progressive hearing loss, left-sided hemiparesis, and impaired coordination. He was hospitalized and reinitiated Biktarvy and PCP prophylaxis with Bactrim. He received broad-spectrum antibiotics for bilateral otomastoiditis and ganciclovir for CMV viremia. Brain MRI revealed findings consistent with progressive multifocal leukoencephalopathy (PML). After discharge, worsening left hemiparesis and new-onset dysarthria led to readmission. A follow-up CT showed progression of subcortical white matter lesions. Lumbar puncture, urine, and plasma were all positive for JC virus. The patient was transferred to the ICU for further management. On day 2, TCCD showed PI of 1.50 on the right and 1.07 on the left, indicating mild asymmetry but no definitive signs of intracranial hypertension. (**A**) MRI exam shows large areas of altered signal intensity, without significant mass effect, hyperintense in 3D axial FLAIR sequence, (**B**) with peripherical diffusion restriction in axial DWI sequence, (**C**) without contrast enhancement in 3D axial T1 FSPGR, involving both cortical and deep regions of the frontal, temporal and parietal regions bilaterally, as well as right insular and thalamic regions. Additionally, the brainstem with left-sided predominance and the right middle cerebellar peduncle are involved. These alterations are consistent with PML. (**D**) MRI perfusion study documents minimal increase in CBF in the more cranial regions on the right. Blue circles indicate the ROI used for quantitative CBF analysis.

**Figure 10:**
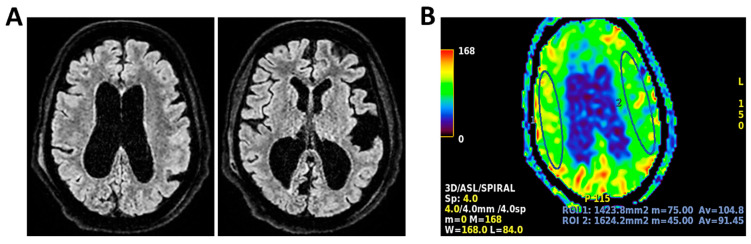
**MRI of patient 5 group 2.** A 67-year-old woman with schizophrenia presented to the ER with lethargy and vomiting, following 10 days of fever and dry cough treated ineffectively with Medrol and Cefditoren. Brain CT showed a left temporoparietal hypodensity (sequela) and supratentorial ventricular dilation without midline shift. The lumbar puncture was positive for *Listeria monocytogenes* (FilmArray). Due to neurological deterioration (GCS 7), she was transferred to the ICU. On day 2, TCCD showed PI values of 1.65 (right) and 1.89 (left), indicating increased cerebral vascular resistance without clear signs of intracranial hypertension. (**A**) 3D axial FLAIR shows marked and diffuse atrophy of the brain tissue, more pronounced in the left hemisphere where some areas of enlargement of the cerebrospinal fluid spaces are observed, more prominent in the parietal region, and diffuse dilation of the supraventricular system, particularly in the occipital horns. (**B**) Perfusion study reveals minimal asymmetry in cerebral perfusion values sampled at the level of the semioval centers. Blue circles indicate the ROI used for quantitative CBF analysis.

All were admitted to the ICU with neurological syndrome caused by suspected or confirmed infectious meningoencephalitis and underwent both TCCD within 48 h of neurological deterioration (Table 1) (GCS < 9), and brain MRI. Patients were divided into two groups based on the type of MRI performed: Group 1 (standard MRI) (Table 2 and Figure 1, Figure 2, Figure 3, Figure 4 and Figure 5) and Group 2 (perfusion MRI with CASL technique) (Table 3 and Figure 6, Figure 7, Figure 8, Figure 9 and Figure 10). A full set of MRI images for each included patient is available in Figure 1, Figure 2, Figure 3, Figure 4, Figure 5, Figure 6, Figure 7, Figure 8, Figure 9 and Figure 10, illustrating the neuroradiological findings in detail. Table 1 reports the following patient parameters recorded during the TCCD diagnostic assessment phase: age, sex, Hct, arterial PaCO_2_, sedation and MAP.

### 3.1. Group 1: TCCD and Standard MRI (FLAIR and DWI)

In the first group, a total of five patients were included. The mean age was 56.4 years (median 49, range 37–76), and four (80%) were female.

Regarding etiology, two cases, case 1 and 5 (40%), were due to pneumococcal meningitis, while the remaining were neurotoxoplasmosis (*n* = 1, 20%) (case 2), Varicella-Zoster virus meningoencephalitis (*n* = 1, 20%) (case 3), and meningoencephalitis of undetermined origin (*n* = 1, 20%) (case 4). Three out of five patients demonstrated normal or mildly elevated pulsatility indices (PI ≤ 1.18) and no waveform signs of intracranial hypertension. In these cases, MRI findings were either normal or showed non-specific alterations, such as mild pachymeningeal enhancement or limited subcortical lesions, without mass effect or restricted diffusion. All three patients in this subgroup experienced full neurological recovery at 28 days (GOS 5) (Table 2 and Figure 1, Figure 2 and Figure 3).

In contrast, two patients in the same group presented with elevated PIs. One patient with HIV and neurotoxoplasmosis exhibited bilateral flow abnormalities (PI 1.96 on the right; 1.25 on the left), with multiple supratentorial ring-enhancing lesions on MRI, suggestive of cerebral toxoplasmosis. The patient showed no significant clinical improvement at 28 days (Table 2 and Figure 4).

Another patient with Varicella-Zoster meningoencephalitis had a critically elevated left-sided PI (3.6) and MRI findings limited to hippocampal hyperintensity (Table 2 and Figure 5).

Despite subtle structural abnormalities, the patient died within 28 days of admission. In both cases, high PI values accompanied poor neurological outcomes, highlighting the prognostic role of TCCD in identifying impaired cerebrovascular reserve and autoregulation failure.

### 3.2. Group 2: TCCD and Perfusion MRI with ASL

In the second group five patients were included. The mean age was 55.4 years (median 63, range 35–67), and three (60%) were male.

Etiological diagnoses were heterogeneous: meningococcal meningitis (*n* = 1, 20%) (case 1), PML (*n* = 1, 20%) (case 2), *Listeria monocytogenes* meningoencephalitis (*n* = 1, 20%) (case 3), meningoencephalitis of undetermined origin (*n* = 1, 20%) (case 4), and pneumococcal meningitis (*n* = 1, 20%) (case 5). Patients underwent MRI with ASL to assess cerebral perfusion. Three patients had normal TCCD findings (PI ≤ 1.4) and symmetrical CBF (differences <15% between hemispheres). ASL imaging confirmed the absence of perfusion deficits, even in cases where FLAIR imaging revealed mild inflammatory changes. All three patients recovered their baseline neurological function at 28 days (Table 3 and Figure 6, Figure 7 and Figure 8).

The remaining two patients in this group experienced poor outcomes. One was diagnosed with progressive multifocal leukoencephalopathy (PML) due to JC virus and presented with a right-dominant perfusion asymmetry and elevated right-sided PI (1.50). MRI revealed extensive subcortical and infratentorial lesions, including the brainstem (Table 3 and Figure 9).

The second patient, affected by *L. monocytogenes* meningoencephalitis, showed bilateral elevation of PI (1.65 and 1.89) and significant CBF asymmetry (right CBF: 105 mL/100 g; left CBF: 91 mL/100 g) (Table 3 and Figure 10).

Both patients died by day 28. Notably, these two cases demonstrated a mismatch between regional perfusion and vascular impedance, consistent with cerebrovascular dysregulation and loss of autoregulation.

### 3.3. Summary of Key Findings

Across both groups, normal PI values (≤1.25) were consistently associated with favorable outcomes, even when inflammatory MRI findings were present. In contrast, elevated PI (>1.25)—particularly when accompanied by asymmetric or pathologic CBF—were related to poor outcomes, including death in all patients with PI > 1.65. These associations suggest that TCCD may function as a dynamic prognostic tool in the early phase of meningoencephalitis, complementing the anatomical data provided by MRI.

Moreover, in the subset of patients with perfusion imaging, the combination of normal PI and symmetric CBF was associated with a full neurological recovery (3/3), while the combination of elevated PI and asymmetric or abnormal CBF was associated with a poor outcome (2/2), underscoring the potential additive value of multimodal non-invasive neuromonitoring. Outcomes in the intermediate PI range (1.25–1.65) were heterogeneous: two patients with maximum PI values of 1.32 and 1.40 had favorable recovery, whereas one patient with maximum PI of 1.50 had poor outcome. This overlap suggests that intermediate PI values may require contextual interpretation (e.g., lateralization, ASL symmetry, and serial changes) rather than a single dichotomous threshold.

As an exploratory analysis in the ASL subgroup (*n* = 5), mean PI showed a positive association with percent CBF asymmetry (Spearman ρ ≈ 0.90). However, this correlation did not reach statistical significance at the two-tailed α > 0.05 level, reflecting the limited statistical power inherent to such a small sample. While the magnitude of the association is consistent with the hypothesis that higher vascular impedance may accompany greater perfusion imbalance, this result should be considered hypothesis-generating only and interpreted with caution.

Additionally, all patients who survived at 28 days underwent follow-up at 3 months, and all had fully recovered with a GOS-E score = 8.

## 4. Discussion

This study highlights the potential of TCCD as a bedside tool to assess cerebrovascular function and stratify neurological risk in adult ICU patients with meningoencephalitis. While MRI remains the gold standard for structural diagnosis, TCCD provides real-time, functional information on cerebral hemodynamics that can complement radiological findings, especially in unstable or sedated patients. Our findings suggest that PI, a surrogate marker of cerebrovascular resistance and autoregulation, may be a sensitive indicator of prognosis: in our cohort of patients, those with PI ≤ 1.25 had favorable neurological outcomes regardless of MRI findings, whereas elevated PI values (>1.25) were associated with poor outcomes, with PI > 1.65 being uniformly related to patient death. Elevated PI likely reflects a final common hemodynamic phenotype: higher downstream resistance and/or reduced compliance, potentially arising from different mechanisms depending on etiology. In bacterial meningoencephalitis, diffuse edema, impaired autoregulation, and raised ICP may contribute; in viral encephalitis, vasculitis or vasospasm may play a role; in opportunistic infections and demyelinating/inflammatory syndromes, microvascular dysfunction and regional perfusion disturbances may be more prominent. In this pilot small cohort study, mechanistic inferences remain speculative and warrant dedicated study with serial TCCD, standardized sedation/PaCO_2_ control, and complementary biomarkers/imaging. Hence, these results are in line with previous studies suggesting that elevated PI reflects microvascular dysfunction, impaired autoregulation, or rising intracranial pressure [15,16,17,18,19,20,21,22]. Importantly, in our cohort, increased PI values frequently preceded structural MRI changes, indicating that TCCD may detect functional compromise earlier than conventional imaging. Moreover, the repeatability of TCCD allows for dynamic assessment over time, enabling the detection of subtle hemodynamic changes that may precede clinical deterioration.

In patients who underwent perfusion MRI with ASL, a strong correlation emerged between CBF symmetry and clinical outcomes. Full neurological recovery was observed in all patients with symmetrical CBF and normal PI, while those with asymmetric perfusion values greater than 15% and high PI succumbed to disease. This supports the hypothesis that the combination of high vascular resistance and regional perfusion deficits represents a critical loss of autoregulatory control—a hemodynamic profile often seen in terminal stages of neuroinflammatory syndromes. From a pathophysiological perspective, meningoencephalitis leads to endothelial dysfunction, disruption of the blood–brain barrier, and impaired vasoreactivity, particularly in vulnerable regions such as the hippocampus, thalami, and brainstem [23,24]. Through continuous PI monitoring, TCCD may offer a non-invasive and repeatable means to detect these alterations in cerebral hemodynamics, but further evidence is needed.

Clinically, these findings suggest several practical implications: (i) TCCD should be considered a first-line neuromonitoring tool in patients with suspected CNS infections, especially when MRI is delayed or not feasible; (ii) serial PI monitoring could enable earlier identification of patients at risk of neurological deterioration, even before structural progression is detectable; and (iii) in centers equipped with ASL, the integration of TCCD and perfusion MRI may improve prognostic accuracy and help tailor therapeutic interventions. The need for standardized multimodal neuromonitoring protocols in meningoencephalitis is clear: combining TCCD-derived hemodynamic indices with structural (FLAIR/DWI) and perfusion data (ASL) could support more comprehensive and individualized care strategies.

We acknowledge that multimodal approaches combining TCCD with other monitoring techniques have already been investigated in various neurological conditions. However, meningoencephalitis—and encephalitis in particular—remains relatively underrepresented within the field of neuromonitoring when compared to other acute brain disorders. Our report adds to the literature by highlighting the potential role of the inflammatory component in meningitis, as suggested by the descriptive MRI findings in both pneumococcal and meningococcal cases, and by drawing attention to possible meningeal inflammatory involvement in presentations with a more encephalitic profile.

Nonetheless, this study has several limitations. It is a retrospective, single-center cohort study with a small sample size, which limits the generalizability of the findings. The retrospective and observational design precludes causal inference, and the heterogeneity in infectious etiologies and imaging protocols further constrain interpretation. Selection bias is likely, as inclusion required availability of both TCCD and MRI (CASL in a subset), potentially enriching for more complex or diagnostically uncertain cases; generalizability to unselected ICU meningoencephalitis populations is limited. In this preliminary cohort, bedside TCCD proved feasible and provided relevant information on cerebral hemodynamics in patients with meningoencephalitis. The observed associations, such as the link between elevated PI and adverse outcome, should be regarded as exploratory and hypothesis-generating, given the small sample size and study design. Similarly, the proposed pathophysiological mechanisms are intended as plausible interpretations rather than proven conclusions.

This preliminary analysis, based on the subset of patients who underwent both TCCD and MRI-CASL, highlights the feasibility and potential value of bedside ultrasonographic neuromonitoring in meningoencephalitis. While the limited number of cases may raise concerns of selection bias, these findings underline the need for further studies to evaluate repeatable and potentially continuous monitoring strategies. Such approaches may provide novel insights into the pathophysiology of cerebral inflammation and contribute to the future management of conditions that are currently underexplored. Larger, prospective, ideally multicenter studies with consecutive enrollment are needed to validate PI ranges across etiologies, to model serial PI trajectories, and to test whether integration with ASL-derived perfusion improves risk stratification and clinical decision-making. In a multimodal framework, the combination of elevated PI and relevant ASL perfusion asymmetry may identify patients with impaired cerebrovascular regulation who need prompt reassessment of modifiable systemic determinants of cerebral perfusion (e.g., MAP targets, PaCO_2_, oxygenation, anemia, temperature, sedation) and consideration of escalation strategies (repeat neuromonitoring, earlier imaging, and invasive ICP monitoring in selected patients). These implications remain conceptual and require prospective testing. We hope that exploring a monitoring approach that is repeatable, potentially continuous, and bedside could, in the near future, lead to new strategies for managing inflammatory brain conditions that have received little attention so far.

## Figures and Tables

**Table 1:** Patient characteristics at the moment of TCCD diagnostic assessment.

				Parameters at the Time of the Diagnostic Assessment			
	Case n.	Age ^a^	Sex	Hct (%)	Arterial CO_2_ (mmHg)	Sedation	MAP
Group 1	1	74	F	36	37.2	Deep sedation with propofol	75
	2	37	F	35	38.4	Deep sedation with propofol	79
	3	49	M	38	35.7	Deep sedation with propofol	70
	4	46	F	39	36.6	Deep sedation with propofol	85
	5	76	F	35	38.1	Deep sedation with propofol	89
Group 2	1	63	M	37	35.2	Deep sedation with propofol	83
	2	45	M	38	39.3	Deep sedation with propofol	76
	3	67	F	38	36.9	Deep sedation with propofol	88
	4	35	M	36	37.5	Deep sedation with propofol	74
	5	67	F	39	35.4	Deep sedation with propofol	81

^a^ Mean age 55.9, (IQR 45.5–68.5).

**Table 2:** Group 1: TCCD and Standard MRI (FLAIR and DWI).

Case n.	Admission		Comorbidities	Diagnosis	Admission EEG						TCCD Findings						MRI Findings	GOS	Outcome at 28 Days
											PI		Mean Flow		CBF				
	SOFA	GCS			Background Activity	Voltage	Regularity	Symmetry of Brain Activity	Evoked Potentials	Epileptiform Abnormalities			(cm/s)		(mL/100 g)				
											R	L	R	L	R	L			
1	8	6	-Atrial fibrillation	Pneumococcal meningitis	4–5 c/s	Low	Yes	Yes	Yes	No	0.62	0.71	37.07	46.39	-	-	Multiple bilateral focal areas of altered signal intensity; hyperintense in FLAIR, diffusion restriction, no mass effect. Pachymeningeal enhancement. Ventricular system normal (Figure 1).	5	Full neurological recovery.
			-Ischemic heart disease																
			-Anxiety-depressive syndrome																
			-Hypertension																
			-Hypertension																
2	6	3	None	Meningoencephalitis ndd.	8–9 c/s	Low	No	No	No	Yes	0.89	0.94	30.01	29.21	-	-	Normal (Figure 2).	5	Full neurological recovery.
3	7	12	-Active smoker	Pneumococcal meningitis	3 c/s	Low to moderate	Yes	Yes	Weak	No	1.18	1.02	17.08	18.06	-	-	No abnormal enhancement post-contrast. Mild diffuse pachymeningeal enhancement of no clinical significance (Figure 3).	5	Full neurological recovery.
4	9	3	-Drug addiction	Neurotoxoplasmosis	3–4 c/s	Low	Yes	Yes	No	No	1.96	1.25	27.85	14.05	-	-	Multiple heterogeneously hyperintense lesions in long TR sequences, one with ring enhancement. Right hemisphere predominant. No mass effect or significant edema (Figure 4).	3	Partial neurological recovery.
			-Alcohol abuse																
			-Tuberculosis in 2018																
			-Active smoker																
5	9	3	-Type I diabetes	Varicella-Zoster meningoencephalitis	3–4 c/s	Low	Yes	Yes	No	No	3.6	ND	18	ND	-	-	Subtle hyperintensity in the right hippocampal region, likely reactive inflammation. No diffusion restriction or contrast enhancement (Figure 5).	1	Death.
			-Hypertension																

ND, not determined. SOFA, sequential organ failure assessment. EEG, Electroencephalogram. R, right. L, left.

**Table 3:** Group 2: TCCD and Perfusion MRI with ASL.

Case n.	Admission		Comorbidities	Diagnosis	Admission EEG						TCCD Findings						MRI Findings	GOS	Outcome at 28 Days
											PI		Mean Flow		CBF				
	SOFA	GCS			Background Activity	Voltage	Regularity	Symmetry of Brain Activity	Evoked Potentials	Epileptiform Abnormalities			(cm/s)		(mL/100 g)				
											R	L	R	L	R	L			
1	10	3	-Hypertension	Meningococcal meningitis	5–6 c/s	Low to moderate	Yes	Yes	Weak	No	1.1	1.4	28	27	61.39	61.97	Areas of altered signal intensity, hyperintense on long TR sequences, with diffusion restriction and high apparent diffusion coefficient, indicative of subacute inflammatory processes (Figure 6).	5	Full neurological recovery.
			-Dyslipidemia																
			-Previous pulmonary lobectomy																
2	8	3	None	Meningoencephalitis ndd	5–6 c/s	Low	No	No	No	Yes	1.32	1.23	28.1	29.3	36.15	38.86	Normal (Figure 7).	5	Full neurological recovery.
3	6	14	-Hypertension	Pneumococcal meningitis	6–7 c/s		Yes	Yes	Yes	No	1.2	1.1	37.1	38.3	44.75	44.59	Normal (Figure 8).	5	Full neurological recovery.
			-Depressive disorder																
			-Hypothyroidism																
4	5	8	-HIV	Progressive multifocal leukoencephalopathy	7–8 c/s	Low to moderate	No	Yes	Yes	No	1.5	1.07	34	47.9	48.3	55.8	Areas of altered signal intensity, hyperintense on long TR sequences, no mass effect (Figure 9).	1	Death.
			-Previous pneumocystosis																
5	5	7	-History of schizophrenia	*Listeria monocytogenes* meningoencephalitis	5–6 c/s	Low	Yes	Yes	Yes	No	1.65	1.89	31.3	22.2	105	91	Minimal asymmetry in cerebral perfusion values (Figure 10).	1	Death.

## Data Availability

The datasets generated and analyzed during the current study are not publicly available. Data will be made available upon reasonable request from the corresponding author and with appropriate ethical approval due to patient confidentiality.
